# Rapid methods for multi-level dissemination of neutralizing monoclonal antibody treatment for COVID-19 outpatients: designing for dissemination using the fit to context framework

**DOI:** 10.3389/fpubh.2024.1412947

**Published:** 2024-11-21

**Authors:** Bethany M. Kwan, Chelsea Sobczak, Lindsey E. Fish, Adit A. Ginde, Gillian Grant, Mika K. Hamer, Kyle Leggott, Vanessa Owen, Jenna Reno, Justin Shrader, Lindsey Whittington, Jenn L. Jones, Ramona Koren, Joel A. Begay, Vall Vinaithirthan, Hillary D. Lum

**Affiliations:** ^1^Department of Emergency Medicine, University of Colorado School of Medicine, Aurora, CO, United States; ^2^Department of Family Medicine, University of Colorado School of Medicine, Aurora, CO, United States; ^3^Colorado Clinical & Translational Sciences Institute, University of Colorado Anschutz Medical Campus, Aurora, CO, United States; ^4^Adult & Child Center for Outcomes Research & Delivery Science, University of Colorado Anschutz Medical Campus, Aurora, CO, United States; ^5^Department of General Internal Medicine, Denver Health and Hospital, Denver, CO, United States; ^6^Division of General Internal Medicine, University of Colorado School of Medicine, Aurora, CO, United States; ^7^Trailhead Institute, Denver, CO, United States; ^8^Center for Bioethics and Humanities, University of Colorado Anschutz Medical Campus, Aurora, CO, United States; ^9^Center for Communication and Engagement Research, RTI International, Research Triangle Park, NC, United States; ^10^Colorado Health Institute, Denver, CO, United States; ^11^Advisory Panel Member; ^12^Lifecourse Epidemiology of Adiposity & Diabetes (LEAD) Center, Colorado School of Public Health, University of Colorado Anschutz Medical Campus, Aurora, CO, United States; ^13^Division of Geriatric Medicine, Department of Medicine, University of Colorado School of Medicine, Aurora, CO, United States

**Keywords:** designing for dissemination, rapid research methods, monoclonal antibody treatment, COVID-19, community engagement, participatory co-design

## Abstract

**Introduction:**

Throughout the COVID-19 pandemic, there was an urgent need for the rapid and equitable translation of knowledge and effective treatments to reach vulnerable populations in response to the ever-shifting pandemic environment. The approval of neutralizing monoclonal antibodies (mAbs) for treatment of outpatient COVID-19 resulted in a need to rapidly design dissemination strategies to increase awareness and equitable access for community members and healthcare providers.

**Materials and methods:**

We used the Fit to Context (F2C) Framework for Designing for Dissemination and Sustainability to (a) design products such as messages and materials, and (b) disseminate the products. We leveraged existing partnerships (e.g., community members, health system leaders, Regional Health Connectors, public health agencies, policymakers, and others) for activities including (a) advising on contextual implementation challenges and opportunities; (b) convening a stakeholder advisory panel; (c) rapid feedback on product reach and impact; and (d) serving as potential product adopters and distributors. We used concurrent data collection and co-design with rapid, iterative prototyping. We used real-world data to evaluate impact of D&I strategies on mAb use in Colorado.

**Results:**

Moving through the F2C Framework phases, we assessed mAb implementation and access barriers and facilitators, identified partner priorities, co-designed messages and materials for multiple audiences, and disseminated through audience-specific communication channels. An emphasis on equity led to tailoring materials to communities with lower health literacy, under- and uninsured groups, Spanish-speaking communities, Native American communities, and rural areas. Dissemination messages, materials, and distribution strategies were updated frequently based on emerging data on COVID-19 treatment effectiveness and availability. Real-world data revealed more than 400% increase in both referrals and number of unique referring providers, with the greatest impact on underserved communities. This was accomplished in under 9 months.

**Conclusion:**

The Fit to Context Framework for Designing for Dissemination and Sustainability is a novel process framework that can inform a rapid, iterative dissemination strategy. The COVID-19 pandemic presented an opportunity to learn better ways to speed translation of evidence to practice and enhance equitable access to evidence-based care. The mAb Colorado project demonstrated the importance of having strong community-academic-public health partnerships and leveraging existing capacity to enhance adoption and reach.

## Highlights

- Methods for Designing for Dissemination and Sustainability (D4DS) include context assessment, application of dissemination and implementation theories and frameworks, and participatory co-design.
- This paper describes rapid methods for application of the Fit to Context Framework for D4DS to development of dissemination strategies intended to enhance equitable access to treatment for COVID-19.
- In 9 months, we developed and enacted successful dissemination strategies for outpatient COVID-19 treatment by leveraging existing multi-sector partnerships, mixed methods context assessment based on diffusion of innovation theory, and co-design with advisory panels and community engagement studios.

## 1 Introduction

From the first clinical reports of the SARS-CoV-2 virus in late 2019 to development of vaccines and therapeutics for prevention and treatment of COVID-19 within the next year, the pace of scientific advancement in response to the COVID-19 pandemic was unprecedented. There was an urgent need for rapid translation of emerging evidence into practice—and thus a role for dissemination and implementation (D&I) science in the pandemic (1). For example, neutralizing monoclonal antibodies (mAbs)—the first effective outpatient therapy for treatment of COVID-19 authorized by the US Food and Drug Administration (2)—became available in November 2020, based on data demonstrating effectiveness in reducing hospitalization and death from COVID-19 (3). Primarily an intravenous treatment, mAbs required a referral from a health care provider and was authorized only for high-risk patients within 10 days of experiencing COVID-19 symptoms. Uptake was slow and less than 5% of available doses were used during the 2020–2021 winter surge (4). Furthermore, there were significant racial/ethnic and geographic disparities in access to mAbs (5). Thus, there was an urgent need for a rapid solution to address low uptake to evidence-based practice and inequitable access to care – which are priority goals and strengths of dissemination and implementation (D&I) science (6).

Four key concepts in D&I were especially relevant to promoting uptake at the health system and provider level and timely, equitable patient access to COVID-19 therapeutics: (a) designing for dissemination and sustainability (7), (b) enhancing the speed of translation to practice (8), (c) attention to the intersection between D&I and health equity (9, 10), and (d) adaptation in response to dynamic context (11, 12). Designing for dissemination and sustainability (“D4DS”) refers to the process of ensuring the products of research align with the needs, characteristics, perspectives, and communication preferences of the intended audience in the setting of expected use (7). D4DS includes the need for dissemination planning, “an active approach of spreading evidence-based interventions to the target audience via determined channels using planned strategies” (13). Planning for active dissemination of evidence is critical, rather than relying on spread through passive diffusion (14). D&I science increasingly attends to health equity and the potential to mitigate disparities by engaging diverse communities and partners at every stage (15, 16).

**Figure 1:**
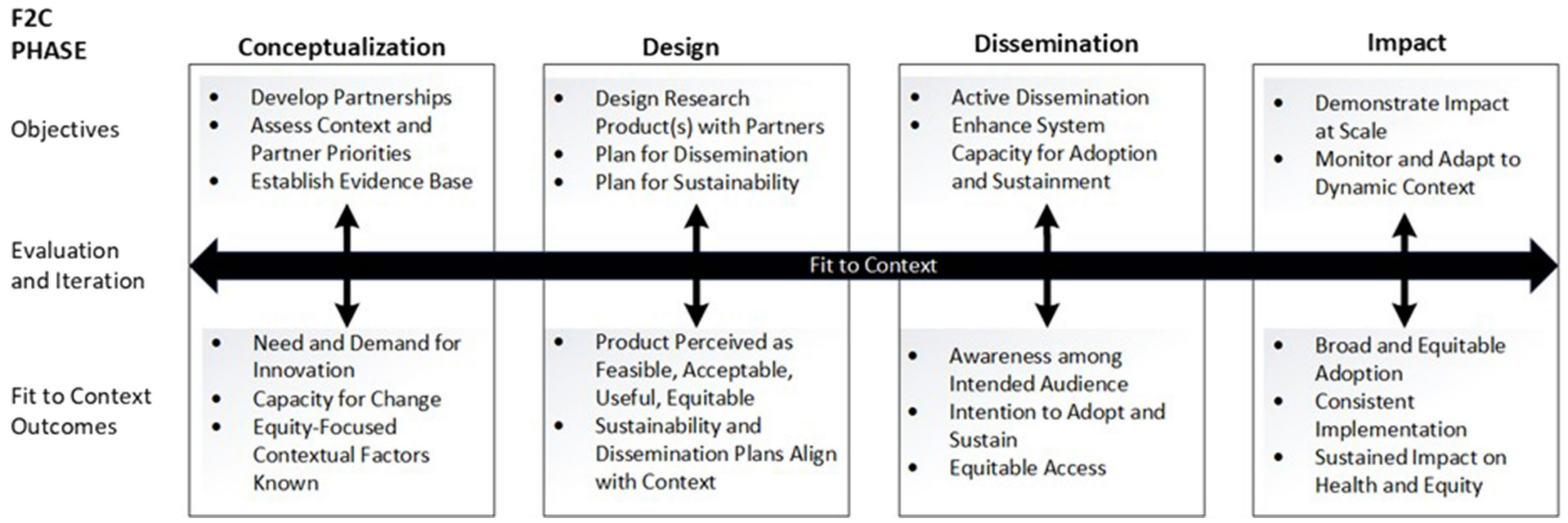
Fit to context (F2C) framework for designing for dissemination and sustainability [Reproduced with permission from Oxford University Press, from the publishers of the Dissemination and Implementation Research in Health, 3rd edition (17)].

This paper describes D4DS methods used to rapidly develop, deploy, and evaluate a multi-level dissemination strategy to increase equitable access to mAb treatment for COVID-19 in Colorado. This was a 9-month academic-public partnership project that took place in 2021, hereafter referred to as “mAb Colorado.” We report the methods and results in alignment with the phases of the novel Fit to Context Framework (F2C Framework) for Designing for Dissemination and Sustainability (Figure 1), which was introduced in its current form in 2023 (17). The F2C Framework is a four-phase process framework that integrates well-established dissemination theory (diffusion of innovations theory as applied to health care (18)) and Bauman and colleagues’ 6-step dissemination planning framework (19). The mAb Colorado project was originally conceptualized by the developers of the F2C Framework, in which plans to design a mAb dissemination strategy included four high-level process steps: (1) stakeholder engagement, (2) context and situation analysis, (3) messaging, packaging, and distribution of dissemination and implementation materials, and (4) adaptation and tailoring. The published version of the F2C Framework (17) is based on a designing for dissemination schema first described in a narrative review of the literature published in the Annual Review of Public Health in 2022 (7) and refinements based on our experience with the mAb Colorado project.

We describe methods used to conceptualize, design, disseminate, and evaluate impact of a multi-level dissemination strategy for mAbs for COVID-19, organized according to the phases of the F2C Framework for D4DS (17). The F2C Framework first includes a conceptualization phase, which starts with (a) mixed methods assessment of contextual factors based on diffusion of innovation theory (20) and (b) convening and understanding partner priorities. Notably, there was a need to understand both clinician and community factors pertaining to awareness of evidence and experience with treatment access systems and policies (21–23). This first step in D4DS – understanding context, barriers, and facilitators to evidence translation prior to designing dissemination strategies – can take considerable time for rigorous data collection, analysis, and interpretation. Next, in the F2C design phase, participatory and co-design methods (17) are used to plan a dissemination strategy that align with context identified in the conceptualization phase activities. A multi-level dissemination approach was designed to reach the recipients of treatment (e.g., people with COVID-19), those delivering treatment (e.g., health care providers), and those making policy decisions about treatment cost, distribution, eligibility, and administration (e.g., health systems, public health infrastructure, and leadership). In the F2C dissemination phase, the design phase plans were enacted, and then evaluated in the F2C impact phase. Given the dynamic context of the COVID-19 pandemic, it was also important to iteratively revisit key partners’ perspectives on awareness, access, needs, and local resources and commit to rapid adaptation of D&I strategies. In this paper, we reflect upon the applicability of the F2C Framework phases and usefulness of a range of D4DS methods carried out in a parallel, iterative process for promoting timely, equitable access to mAb treatment. Detailed descriptions of the methods and results for distinct aspects of the project are described elsewhere.

## 2 Methods

### 2.1 Ethics approval and consent to participate

This project was reviewed and approved by the Colorado Multiple Institutional Review Board (COMIRB) under protocols #21-2747 and #21-2872. All methods were conducted in accordance with relevant guidelines and regulations.

### 2.2 Design and overview

The purpose of this project was to conceptualize, design, disseminate, and evaluate impact of a multi-level dissemination strategy for mAbs for COVID-19 in Colorado. We used a variety of D4DS methods (participatory and co-design methods, context analysis, application of D&I frameworks, communication and the arts), as described by Kwan et al. (7). We were continually responsive to changes in context, including COVID-19 epidemiology, mAb treatment agent effectiveness and availability, and federal and state access policies. To speed up the D4DS process for mAb treatment, compared to a standard sequential process, we set an ambitious 9-month timeline (March 2021–December 2021),assembled a large multidisciplinary team funded as a supplement to our institution’s Clinical and Translational Sciences Award (CTSA), and proceeded with the F2C Framework phases in a parallel, iterative manner. Specifically, rather than sequential assessment of barriers and facilitators followed by designing dissemination strategies, we used concurrent data collection and co-design with rapid, iterative prototyping. The research team included expertise in D&I science, participatory research and co-design, health communication, several clinical disciplines, and survey, qualitative, and mixed methods. The research team met at least weekly, with multiple smaller working groups to make progress on discrete tasks and then coming together for integration and synthesis.

**Figure 2:**
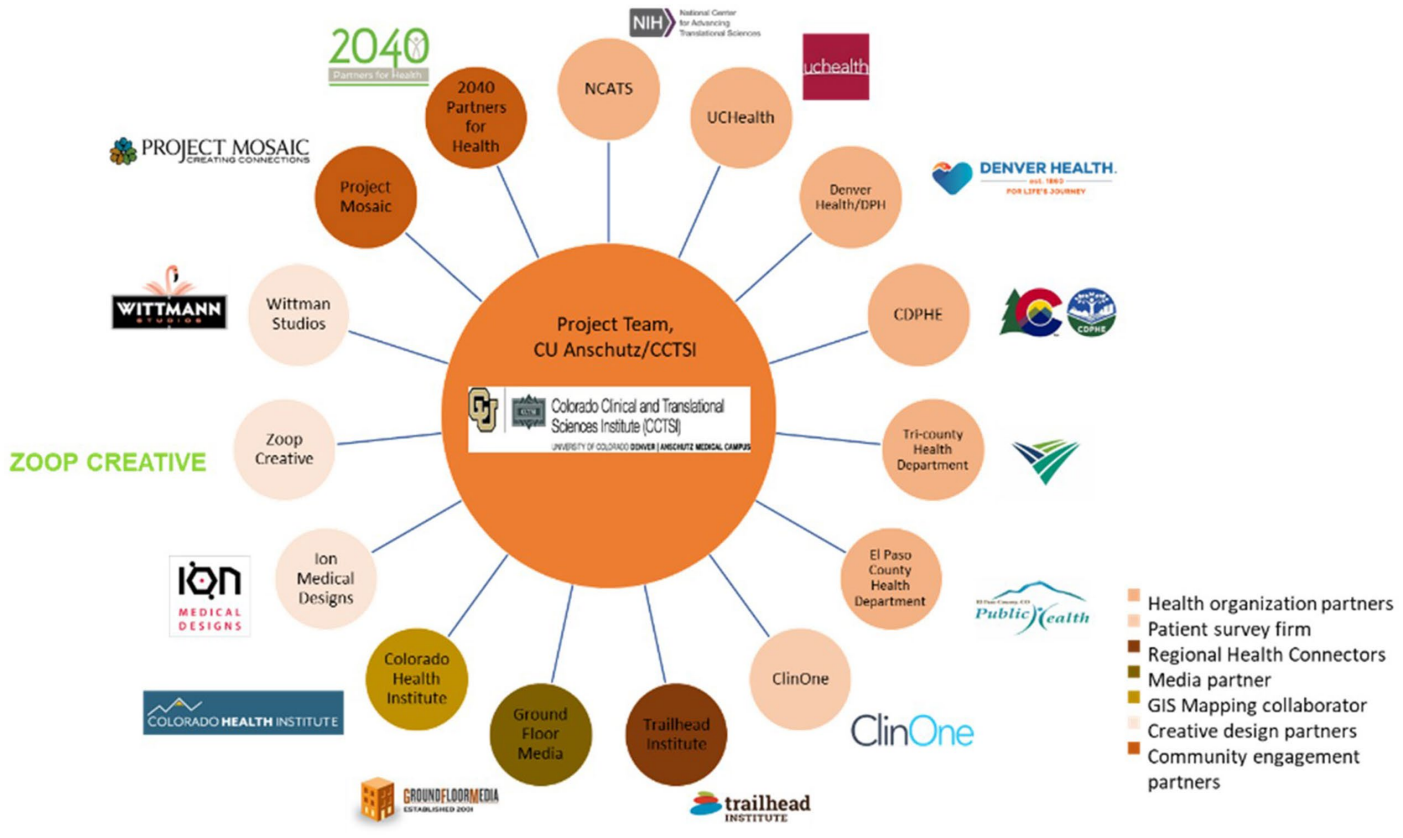
mAb Colorado partners.

We leveraged existing partnerships for both data collection and co-design, relying upon relationships cultivated with community members and organizations over prior years (Figure 2). One essential partnership involved the Regional Health Connectors (RHCs), a locally embedded workforce that aims to improve health in Colorado by connecting health systems with community assets and resources. Organizational partners also included UCHealth (academic health system), Denver Health (safety net health system), Colorado Department of Public Health and Environment (CDPHE; COVID-19 state response agency), local public health agencies (LPHAs), the Trailhead Institute (RHC program support), and the Colorado Health Institute (data and analytics partner). Dissemination strategies were updated frequently as our data emerged and as COVID-19 science and policy evolved, with input from partners. We used real-world data to monitor and evaluate dissemination strategy impact on uptake and use of mAbs in Colorado (24).

### 2.3 Fit to context framework for D4DS

As shown in Figure 1, the F2C Framework for D4DS (17) is a process framework depicting four research phases (conceptualization-design-dissemination-impact) with an explicit focus on dissemination, sustainability, and equitable impact on health. The F2C Framework was developed following a review of the D4DS literature and builds upon D&I logic models, *push-pull-capacity* and diffusion theory concepts, and Bauman’s six-step dissemination planning framework (7). In each F2C phase, there are target outcomes and objectives. There are multiple research methods relevant to co-design, evaluation, and iteration at each phase.

#### 2.3.1 F2C conceptualization phase

F2C Conceptualization Phase target outcomes included demonstrating need and demand for an innovative approach to enhancing mAb treatment access, determining capacity for change, evaluating the evidence base, and identifying equity-focused contextual factors (e.g., disproportionate impact of COVID-19 among minoritized racial/ethnic groups, rural communities, those living in long-term care facilities, and unhoused communities). Conceptualization Phase activities began in March 2021 and continued through December 2021 to allow adaptation to dynamic context (i.e., partner capacity and priorities, health equity gaps, COVID-19 public health impact and evidence). Research team activities included convening a stakeholder advisory panel (henceforth, the advisory panel), establishing regular communication with health systems’ and state public health leaders, assessing context related to mAb awareness [i.e., process mapping, health care provider and community member surveys, interviews, focus groups (21–23)]; and reviewing emerging evidence (i.e., data on treatment efficacy; disparities in COVID-19 epidemiological data, existing national mAb dissemination toolkits). Partner activities included (1) advising on contextual implementation challenges and opportunities; (2) identifying advisory panel members; (3) providing data and rapid feedback related to impact; and (4) implementation and/or distribution of products to target audiences. The advisory panel consisted of 25 people (9 community members; 3 clinicians including 1 family medicine, 1 infectious disease, and 1 long-term care provider; 2 state public health agency representatives; 8 RHCs; and 3 representatives of health equity-focused community-based organizations). The advisory panel met virtually once or twice a month for 1 year (April 2021 – April 2022) to inform collection and interpretation of Conceptualization Phase data, discuss dissemination strategy drafts, prioritize health equity issues and solutions, and plan for academic manuscripts.

#### 2.3.2 F2C design phase

F2C Design Phase target outcomes included demonstrating feasibility and acceptability of the mAb dissemination strategies and identifying distribution channels that aligned with how Colorado community members and health care providers receive information. Design Phase activities occurred between May and November 2021, integrating Conceptualization Phase insights as they emerged. The focus was to iteratively design and refine messages and materials, adopt strategies to address health equity gaps, and plan for dissemination to target audiences. Key activities included engaging communities and partners in co-design of dissemination strategies and employing design specialists (i.e., graphic designer, visual illustrator, web designer, video creators, media specialists).

In addition to ongoing engagement with the advisory panel throughout the Design Phase, we used community engagement studios [CE Studios] (25) to co-design the dissemination strategies. CE studios are a well-established method for community engagement that has been applied to multiple aspects of research study design, including dissemination planning (25). CE studios involve 2–3 group discussions with community representatives, convened in partnership with community partner navigators and led by a neutral facilitator. In a CE Studio focused on dissemination planning, after a short expert presentation on the topic of interest (in this case, mAb evidence and availability), the group is then engaged in discussions about how best to disseminate the evidence to their community (i.e., the messaging, packaging, and distribution channels). In between sessions, the research team creates and refines dissemination products (with support from graphic designers). We conducted nine CE Studio sessions with five Colorado communities to design dissemination products with and for the Native American community (2 virtual sessions), the Hispanic/Latinx community (1 in-person session in English and Spanish), rural communities (2 virtual sessions), urban communities (2 virtual sessions), and health care providers (2 virtual sessions). Given concerns about COVID-19 exposure and to facilitate statewide participation, all were conducted virtually except for one CE Studio with the Hispanic/Latinx community, at their request. The communities selected for engagement through CE Studios were informed by the advisory panel health equity priorities.

#### 2.3.3 F2C dissemination phase

F2C Dissemination Phase target outcomes included increased community awareness of mAb availability, provider intention to adopt mAb referral processes, and demonstration of broad and equitable reach to target audiences. Dissemination Phase activities began in late July 2021 as Design Phase products emerged and evolved in response to dynamic context; active dissemination continued through December 2021. The dissemination strategies focused on reaching multiple audiences to increase awareness about mAb treatments. Target audiences were members of the public (especially rural and racial/ethnic minoritized populations), health care providers (primary care, urgent care, and long-term care settings), health system and public health leaders (including COVID-19 testing sites), and state and county policymakers.

The mAb Colorado partners collaborated to enhance capacity for distribution and adoption of mAb messaging and materials. We met regularly with public health leaders to inform dissemination and implementation of equity-enhancing strategies. We contracted with a digital marketing company to run a paid media campaign using materials produced in the Design Phase, including social media advertisements, radio spots, and direct mail. We assessed awareness and reach among intended audiences using multiple methods and data sources, including a distribution tracker, newsletter metrics, and digital analytics. As part of dissemination to health care providers, we conducted multiple virtual presentations with clinical audiences, including ECHO Colorado, an affiliate of Project ECHO (Extension for Community Health Outcomes) - an existing recognized model for rapid dissemination of medical and public health knowledge to health care providers. The ECHO model has been adopted world-wide as a reliable, effective intervention to address gaps in care and education (26, 27). As part of the ECHO webinars, we surveyed participants’ intention to adopt practices related to mAb referrals for outpatient COVID-19 patients using a subset of items from the Conceptualization phase provider survey (22).

#### 2.3.4 F2C impact phase

F2C Impact Phase target outcomes included increased mAb referrals (per Colorado mAb referral system), and health equity outcomes (per COVID-19 hospitalization and mortality data). Impact phase activities began in May 2021 and continued through December 2021. We requested de-identified, aggregate referral data from November 2020 through the end of December 2021 (when data emerged suggesting reduced efficacy for mAbs as the omicron variant became dominant). Data included total number of referrals and total number of unique referring providers at each of the state’s mAb treatment sites each week. Based on the location of each treatment site, we linked these data to publicly available county-level COVID-19 public health surveillance data and social indicators. The COVID-19 data included weekly COVID-19 case rate, death rate per 100,000 population, hospital bed occupancy rate by suspected and confirmed COVID-19 cases, and percent of the population fully vaccinated (overall and by age group). The social indicators included county-level population percentages by race/ethnic group, non-English language speakers, and those living below the poverty level, as well as a Colorado COVID-19 social distancing index (28).

For rapid monitoring, CDPHE provided approximately monthly referral data, starting in March 2021. We mapped the monthly data using Esri geographic information system software (ArcGIS Pro) and used visual inspection to assess changes in numbers of mAb referrals and unique referring providers across the state over time. This allowed us to assess the need for adaptations to dissemination strategies in response to dynamic context, allowing geographically-targeted messaging. The research team met at least weekly to monitor changes in COVID-19 policy, from changes to the mAb EUAs and payer policies to shifts in the dominant variant and corresponding mAb efficacy (29). At the end of the Dissemination Phase, we requested a complete data set (November 2020 thru December 2021) of weekly aggregated referrals and unique referring providers in Colorado. We gathered publicly available data on mAb dose allocations from the US Department of Health & Human Services’ Administration for Strategic Preparedness and Response (ASPR). The weekly referrals and dose allocation data allowed precise evaluation of impact on mAb adoption. For this impact analysis, we created maps showing the number of mAb referrals per 1,000 COVID-19 positive cases per week by county across five discrete time periods distinguishing phases of the pandemic. The mAb Colorado dissemination strategies were broadly launched beginning at the end of July 2021 and continued throughout fall 2021. We created line graphs of total referrals per week and total number of unique referring providers throughout this time. We used a retrospective cohort study design to evaluate changes in weekly mAb referral rates before and after launch.

## 3 Results

### 3.1 F2C conceptualization phase outcomes

F2C Conceptualization Phase activities demonstrated a need for both provider- and community-focused mAb dissemination strategies. Results of our mixed methods analysis of community members’ awareness and attitudes towards mAb treatment showed few people had heard of mAbs in summer 2021 but most were willing to consider treatment if recommended by a doctor (23). Participants recommended that dissemination of how to get mAb treatment be shared with “everyone everywhere” through multiple channels tailored to local community systems of influence and communication– and that healthcare providers be prepared to field questions from patients about the treatment. The advisory panel emphasized equity-oriented concerns about costs, especially for those who were uninsured, underinsured (e.g., high deductible plans), and access, especially among rural, undocumented, and unhoused communities. In parallel, results of health care provider surveys and interviews showed existing treatment and referral systems relied upon individual providers, who may see only one or two COVID-19 positive patients per month and may not be aware of evolving mAb EUA criteria and treatment sites (21). This suggested that a centralized resource for mAb referrals may be more appropriate than relying upon individual providers to keep up with the latest evidence and process changes. While primary care and emergency department providers felt it was generally feasible, acceptable, and appropriate to oversee the mAb referral process for their patients, finding an available treatment location was a barrier. Accordingly, there was a need for provider education on mAb evidence, treatment processes, and referral systems, as well as enhanced system capacity for treatment across the state. Engaging larger health systems to expand treatment locations and supporting centralized mAb referral systems were seen as key opportunities for capacity building. Health equity priorities included prioritizing access to and building capacity for treatment in rural communities, Hispanic/Latino communities (including monolingual Spanish speaking communities), Native American communities, and people who were uninsured, underinsured (or had high deductible health plans), undocumented, or unhoused.

### 3.2 F2C design phase outcomes

As established in the CESs, community-specific messages and materials were created in English and Spanish. We followed https://www.plainlanguage.gov/ guidance on readability. In line with community priorities, we developed messages that (1) emphasized safety and effectiveness of the treatment, (2) provided general cost information (e.g., medications were free; treatment delivery may have a cost for some), and (3) encouraged people to talk to a health care provider to see if the treatment was right for them. Specific cost information and full cost transparency was unattainable and an ongoing barrier to public communication. Based on advisory panel recommendations, design considerations and adaptations were prioritized to reach Native American community audiences, as well as people with immunocompromising conditions. We partnered with a medical illustration company to create a graphic novel and animated video^1^. Health care and local public health messaging products included a COVID-19 mAb referral checklist, patient eligibility checklist, and an implementation blueprint. An implementation blueprint was designed to support practices in becoming mAb treatment sites and implementing referral processes (30).

The dissemination strategies involved multiple distribution channels, informed by Conceptualization and Design Phase activities regarding trusted messengers and sources of information among intended audiences. Distribution channels included RHC networks, social media, mAb Colorado website promotion, mAb Colorado newsletter, and presentations to clinical and public health audiences. Key suggested channels included ECHO Colorado, Colorado Community Health Network representatives, and COVIDCheck Colorado, a testing company with broad reach throughout the state. During presentations with health care providers, local public health leaders and other practitioners, we received feedback to iteratively refine the dissemination products for the changing context and clinical needs related to mAb. The advisory panel members repeatedly asked mAb Colorado leadership to request that the governor make public statements about mAbs.

Partners recommended several mAb care models to enhance equitable access to treatment, reflecting the recognition that education alone was insufficient and that capacity building was needed. They suggested opportunities to provide equitable access to mAbs through enhancing urgent care-based models, outreaching local homeless shelters, partnering with home health agencies and LPHA, increasing the number of clinical sites offering treatment on site, instituting a statewide call center for the public, enhancing information on the CDPHE website, and deploying mobile treatment buses that would travel to areas of greatest need. There was also interest in allowing patients to self-refer (overcoming the need for a provider to issue a referral) and instituting standing orders. Providers wanted a central referral mechanism to call and find available appointments. In parallel, many partners suggested that case investigators calling people who have tested positive for COVID-19 could connect patients to a central treatment referral mechanism.

### 3.3 F2C dissemination phase outcomes

In the F2C Dissemination Phase, we enacted the Design Phase dissemination strategies and implemented capacity building. Table 1 summarizes distribution and reach. Materials and distribution channels included: (1) social media (Facebook and Instagram) advertisements, (2) Google search advertisements, (3) local radio spots, (4) direct mail, and (5) printed flyers distributed via community partners. mAb Colorado created a website using Sitefinity, the University of Colorado School of Medicine’s content management system and web hosting platform, to house the materials, such as electronic files and a print copy request form (31, 32). The website launched in May 2021 and had an increase in visitors by mid-July 2021, after promotional efforts began, and by February 2022 had 41,689 visitors. The mAb Colorado project sent at least monthly newsletters from July 2021–January 2022, totaling 10 editions.

**Table 1:** mAb message and material distribution channels, metrics, and reach by audience type.

Distribution channel	Metric	Reach
Community products and materials		
mAb Colorado website		
	Unique Users Clicks for mAb Treatment	41,689 (as of February 2022) 1,346
mAb Colorado social media		
Unpaid social media	Impressions (Facebook) Impressions (Instagram) Click Thru Rate (Combined)	7,100 8,000 0.27%
Paid social media	Impressions (Combined) Clicks (Combined) Click Thru Rate (Combined)	8,806,858 23,625 0.58%
Radio with social media and web takeover		
All radio campaigns	Persons Reached	20,235
KQKS	Impressions Clicks (Click Thru Rate)	65,522 103 (0.20%)
KEKB	Impressions Clicks (Click Thru Rate)	2,17,651 318 (0.15%)
KKNN	Impressions Clicks (Click Thru Rate)	21,483 60 (0.10%)
Print materials		
Flyers and postcards	Distributed by Hand	5,080
Direct mail postcards	Mailed	49,079
Graphic novel	YouTube Views	102 (March 2022–May 2023)
	Print Copies Distributed	35
Provider messages and materials		
mAb Colorado E-newsletters		
Monthly and special editions	Total number of newsletters	10
	Number of subscribers	304
	Open rate: average (min-max)	44.0% (41–59%)
	Click rate: average (min-max)	6.9% (4–10%)
Presentations		
Local public health	Presentations	4
	Estimated total attendees	113
Health care clinics	Presentations	6
	Estimated total attendees	195
Academic partners	Presentations	4
	Estimated total attendees	295
Professional organizations	Presentations	7
	Estimated total attendees	892
Project ECHO	Webinars	6 (3 in August 2021, 3 in December 2021)
	Total registrants	2,020
	Live attendees	1,129

* Does not participate in Neilsen Radio tracking and did not provide data for radio spots.

The RHCs were a valuable distribution channel for community and provider messages and materials. RHCs serve as a crucial point of connection in the community, given their relationships with primary care practices, behavioral health care providers, public health and other community organizations. RHCs distributed communication materials monthly from May 2021 through December 2021 about the effectiveness, utility, and how to access mAb treatment. RHCs coordinated 6 educational outreach events about mAb treatment, reaching 165 providers, partners, and community members across the state of Colorado.

In partnership with ECHO Colorado, we delivered a webinar series, “A Provider’s Guide to Monoclonal Antibody Therapy for COVID-19” to Colorado health care providers (MD, DO, APP, RN, etc.) and administrators interested in outpatient mAb treatment for COVID-19 patients. This webinar included both the evidence supporting use of this treatment and strategies for local implementation of mAb referral and treatment processes. The first series in August 2021 was held three times; a second update series in December 2021 was held three times. Across the August and December sessions, there were 2,020 total registrants, of whom 1,129 attended in-person (average 188 participants per session); all registrants received a recording of the webinar, slides, and shared resources.

Data from the ECHO webinars’ polling questions and post-survey questions showed exposure to the presentation increased likelihood of referring patients with early symptomatic COVID-19 who are at high risk for severe illness and hospitalization for mAb treatment from 55 to 81%. Impact score data from ECHO post-survey showed respondents (n = 684, 61% response rate) rated clinical relevance of the content as 4.2 out of 5. In addition, we delivered 24 presentations to other clinical, public health, professional organizations, and academic audiences. In total, we estimate target audiences were engaged in 20+ hours of collective presentation and discussion time, reaching 2000+ individuals.

To build capacity for treatment, referral, and more equitable access to care, the mAb Colorado project supported implementation of several Design Phase care models. First, in partnership with UCHealth, the mAb Colorado team supported expanded capacity for a centralized referral mechanism through the virtual health center (VHC). The VHC includes virtual urgent care (VUC), which offers telehealth-based urgent care services for common conditions including respiratory symptoms (33). The UCHealth VUC shifted resources to take both clinician-initiated and community patient-initiated referral requests for mAbs. VUC providers served as a centralized resource with up-to-date expertise on mAb eligibility criteria and well-learned routines for identifying treatment locations and placing referrals and orders. This resource addressed concerns regarding relying upon individual providers to stay current on treatment eligibility and referral processes, and about the time it would take to find an available appointment.

Additionally, we worked with two LPHAs to pilot a case investigator referral process with the UCHealth VHC. Over 4 months, LPHA case investigators referred 217 people to the VHC for a mAb treatment consultation, with about 60% ultimately receiving treatment. A mAb Colorado team member who directs a Denver Health urgent care clinic established an exemplar urgent care-based COVID test-to-treat program (34). This clinic provided mAb treatment to 2,524 patients over a 17-month period and demonstrated equitable race and ethnic distribution of treatment (7).

In collaboration with the mAb Colorado team and informed by the advisory panel requests, CDPHE implemented several public health infrastructure and policies for facilitating access to mAbs. The main competing demand that limited implementation of recommended policies was prioritization of vaccine messaging and concerns about overwhelming the public with simultaneous information about prevention and treatment. In anticipation of potential treatment shortages, the state’s mAb referral tool was built with the capacity to “randomly allocate” someone to treatment or no treatment. Accordingly, the tool was originally named the mAb Random Allocation tool. This functionality was never activated, and all patients were referred for treatment. Given concerns discovered in provider interviews that this random allocation process was in place, the mAb Colorado team recommended re-naming the tool the “mAb Connector Tool.” Although a minor change in labeling, it was necessary to address a key clinician barrier to mAb referral. Next, the state integrated language about COVID-19 treatment availability in the post-call emails the state’s case investigators sent to positive cases, directing people to the state’s treatment website or to call their doctors for more information. A more substantial policy change, announced by the Governor of Colorado on November 19, 2021, was a public health order that allowed people with COVID-19 to self-refer for mAb treatment (35); the state subsequently set up a website that allowed patients seeking treatment to book appointments online at new state-run treatment centers, including fixed sites and “mobile buses.”

Some proposed models were not able to be implemented or implemented in a very limited capacity. For example, a “Hospital at Home” program concept was not feasible because it required specific approvals that could not be implemented in a timely manner. Similarly, the statewide call center concept was not feasible because people calling would still need to find a provider to issue a referral—such that a call center would represent a potentially inefficient middle step. A planned mAb referral workflow at a Denver Urban Indian Health Program clinic was only partially implemented, as it went live in December 2021 right as mAb usage was declining due to decreased efficacy.

### 3.4 F2C impact phase outcomes

**Figure 3:**
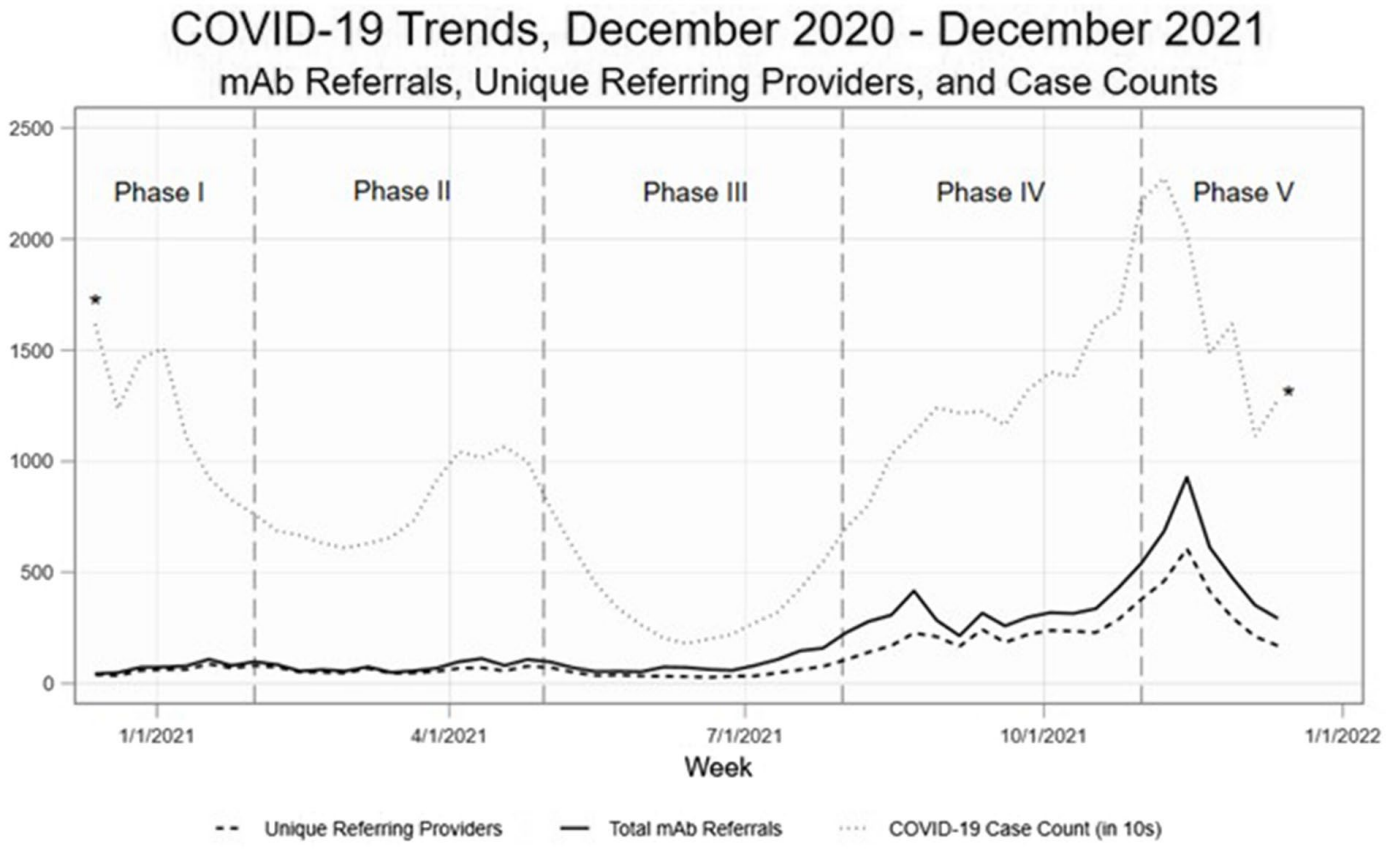
Observational trends, by phase of the pandemic. Phase I. November 2020–January 2021 (initial mAb availability, low uptake), Phase II. February – April 2021 (B.1.1.7 variant surge), Phase III. May – July 2021 (vaccines broadly available, lower transmission rates), Phase IV. August – October 2021 (B.1.617.2 variant surge, temporary mAb shortages), and Phase V. November–December 2021 (B.1.617.2 gives way to B.1.1.529 variant; most mAb EUAs revoked).

**Figure 4:**
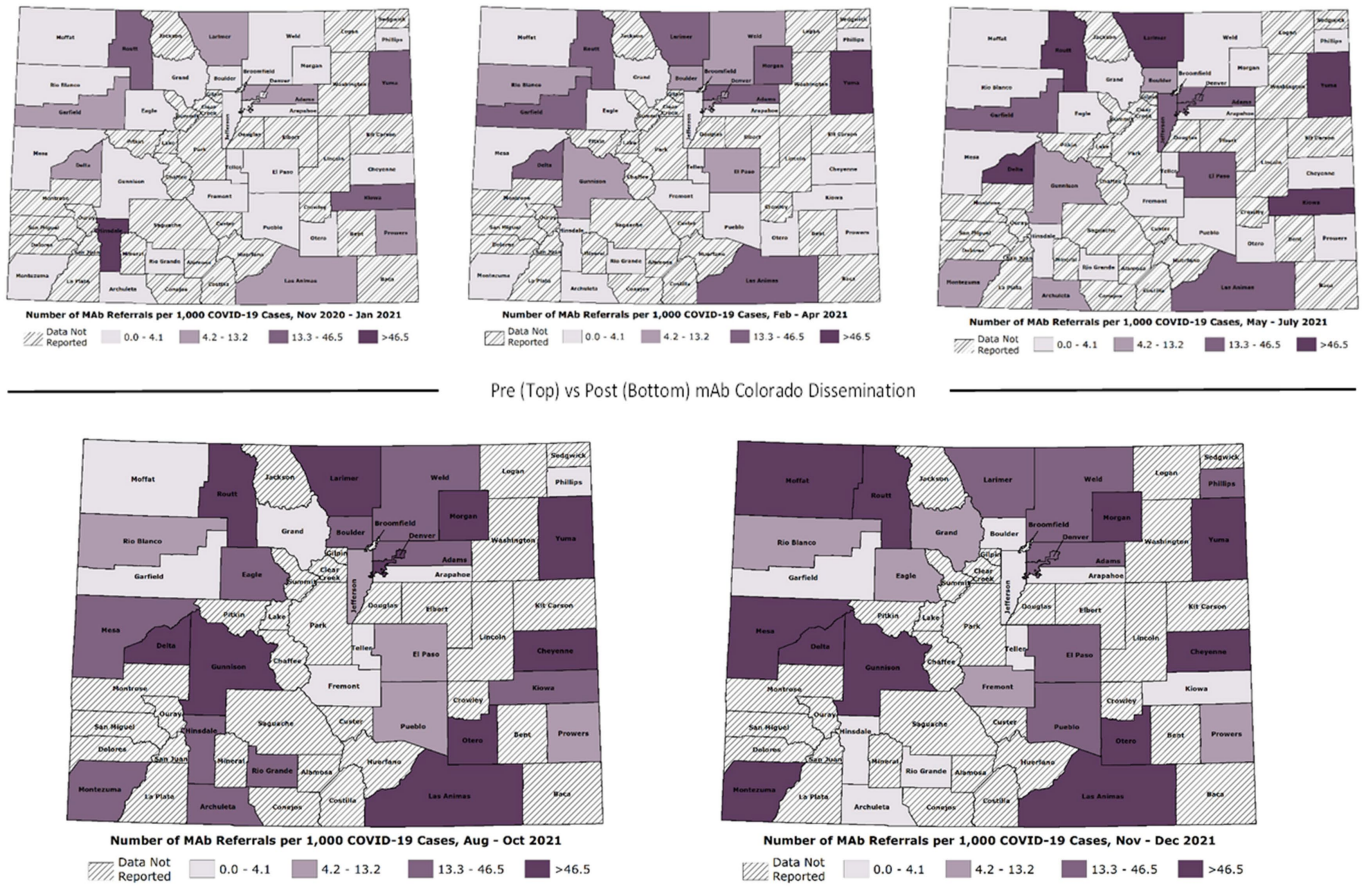
Average weekly mAb referral rates by Colorado county over time (November 2020–December 2021).

**Figure 5:**
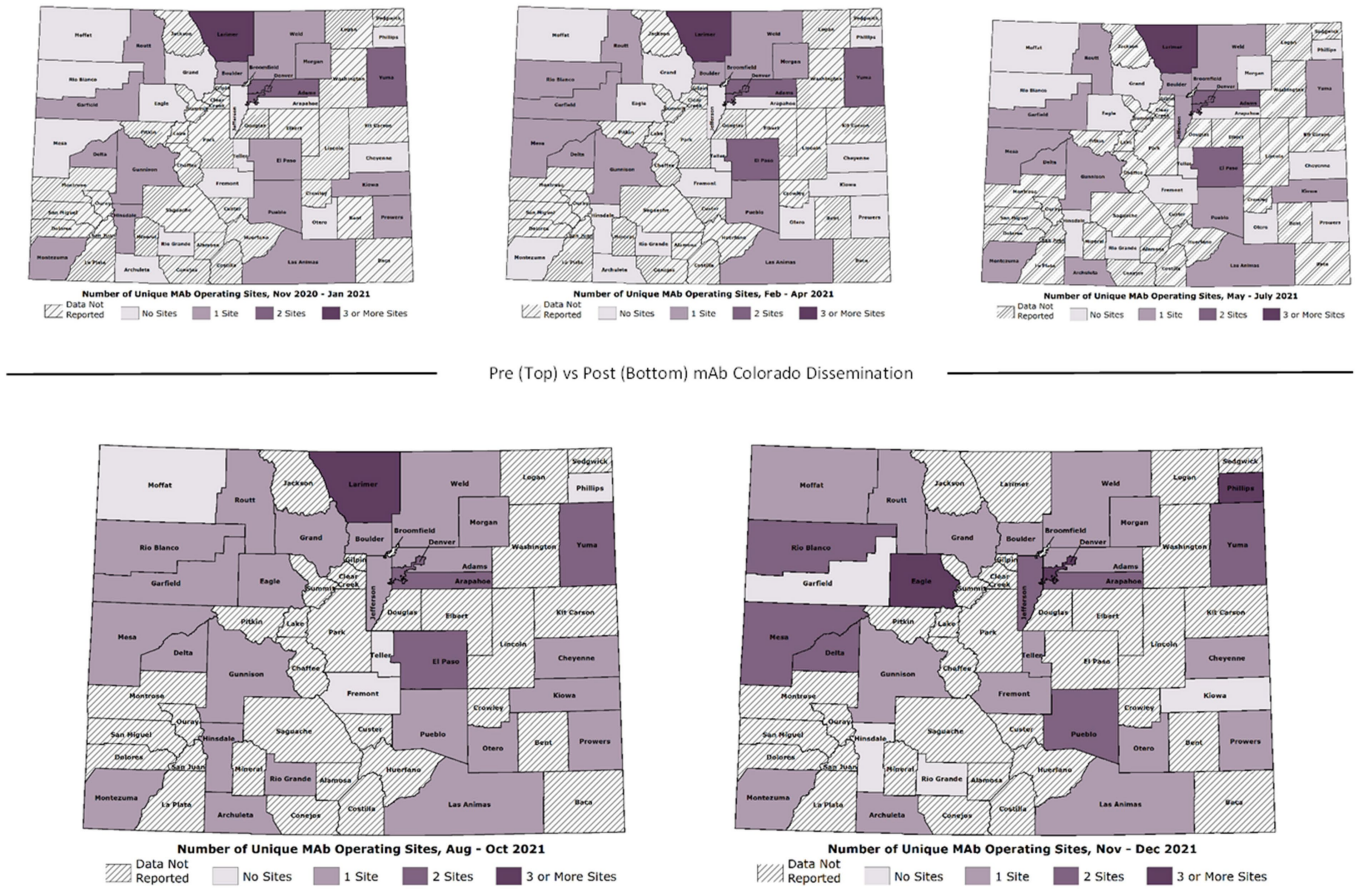
Number of operating mAb treatment sites by Colorado county over time (November 2020–December 2021).

**Figure 6:**
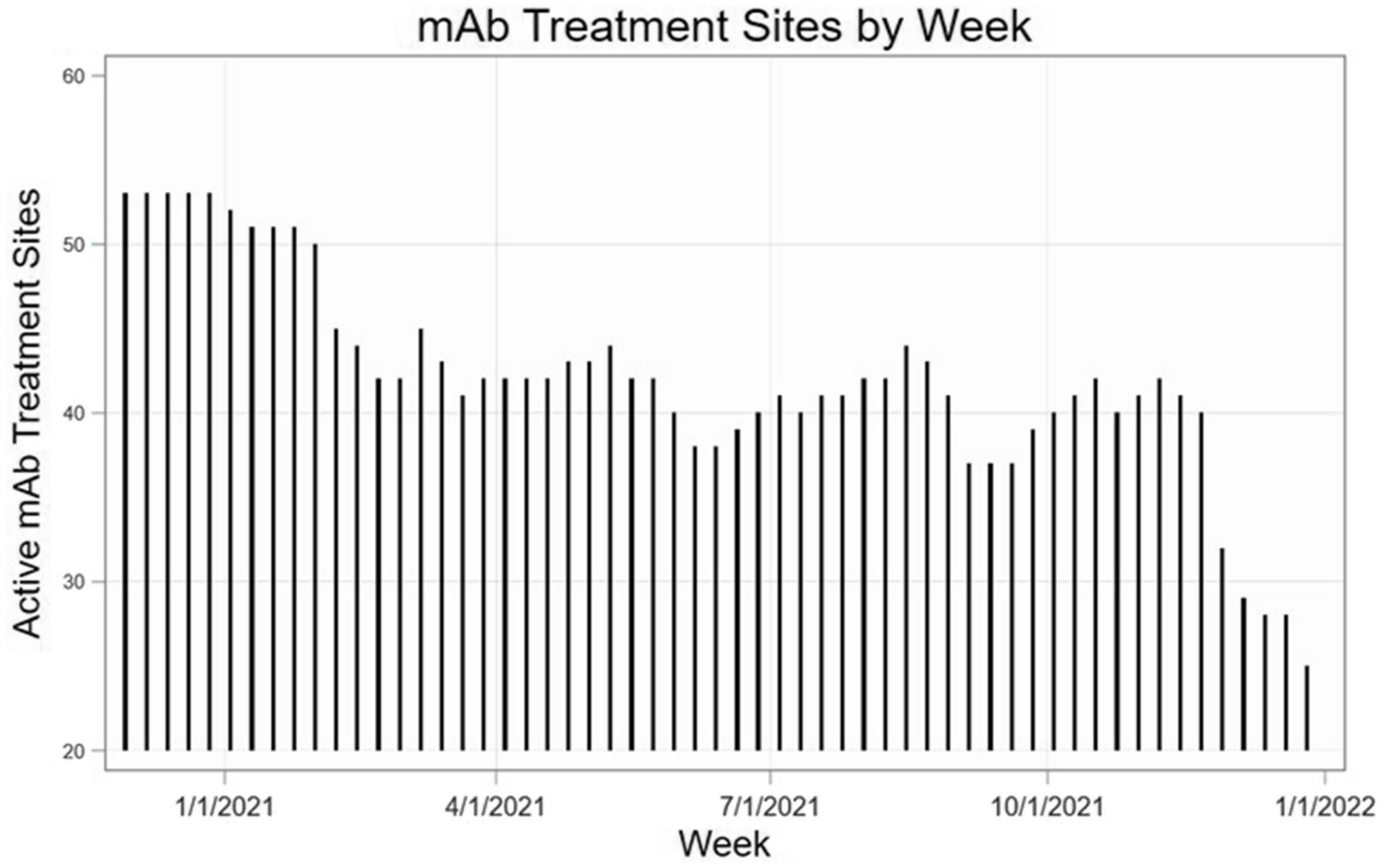
Statewide number of active mAb treatment sites over time (November 2020–December 2021).

As demonstrated in Hamer et al. (24), there were significant changes over time in total mAb referrals and unique referring providers by week after launching the dissemination campaign. Compared to the “pre” period (November 2020–June 2021) before the mAb dissemination plan was enacted, average per-county weekly referrals increased by 417.4 percentage points from 2.99 to 15.47 in the “post” period (July – December 2021; Figure 3). Figures show successive maps of average weekly rate of mAb referrals per 1,000 positive cases of COVID-19 (Figure 4) and number of operating treatment sites (Figure 5) by county over time from November 2020 to December 2021. After initial increases, the total number of mAb treatment sites statewide decreased over time as the doses available became more limited and the only subcutaneous mAb option became ineffective (Figure 6). In addition, the Office of the Governor of Colorado reached out to the mAb Colorado team to connect their office to an individual featured in a testimonial video created as part of the efforts to educate the public that was posted to YouTube. CDPHE also subsequently integrated concepts from mAb Colorado messages into their public health messaging about mAbs, such as mailed postcards statewide (e.g., “Ask your doctor about treatments for COVID-19. If you or a loved one test positive for COVID-19, treatments are available that can help prevent serious illness.”—followed by guidance about timing, effectiveness, where to access treatment, and state contact information).

The F2C Impact phase includes monitoring and adapting in response to dynamic context. The speed of change of science and policy required materials to be updated frequently which was, at times, a limiting factor in providers staying current about treatments and referral processes. In December 2021, *in vitro* data suggested the mAbs available at the time were no longer active against the emerging omicron variant, and the EUAs for all but one mAb agent (sotrovimab) were revoked in January 2022. Sotrovimab was thought to retain efficacy against omicron, although it was in limited supply. Unfortunately, sotrovimab was later shown to be ineffective for omicron (29), particularly after the transition to BA.2 subvariants in March 2022. At the same time, EUAs for several oral antiviral treatments were issued and outpatient treatment for COVID-19 largely transitioned to these oral agents. As dissemination of oral treatments was out of scope for this project, the mAb Colorado project dissemination activities were considered complete as of December 31, 2021.

## 4 Discussion

In this first application of the Fit to Context (F2C) Framework for D4DS (17), we demonstrated a rapid 9-month process of conceptualization, design, dissemination, and impact evaluation of a multi-level dissemination strategy for mAb treatment for COVID-19. The F2C Framework served as a useful and unique approach to guide multi-phase integration of evidence-based approaches to community engagement, participatory co-design, theory-informed dissemination, and mixed methods research. Other frameworks that have emerged in the aftermath of the COVID-19 pandemic to guide rapid D&I in public health emergencies [e.g., the pragmatic, rapid, and iterative dissemination and implementation (PRIDI) cycle (11)] also emphasize the importance of stakeholder engagement, assessing context and the evidence base, and iterative evaluation and adaptation to dynamic context. It is not a new concept in D&I that to impact practice or policy we must “foster collaborative and reciprocal relationships among researchers, implementers, and other stakeholders” to ensure research is relevant, rigorous, rapid and recursive, replicable and clearly define required resources (36). The F2C Framework adds to the sea of D&I frameworks (37) by incorporating a focus on dissemination and sustainability planning, attending to health equity-oriented contextual factors, and building and leveraging existing capacity for delivery of services in complex systems.

It is important to have access to data to inform iterative D&I strategies (38). Throughout this project, we combined access to timely data with interpretation and co-design including community, health system, and public health partners. This included both primary and secondary data collection and analysis. For instance, survey, interview, and focus group data informed tailored messaging to multiple audiences delivered by trusted local, regional, and organizational sources such as healthcare organizations, public health agencies, and media outlets. Access to close-to-real-time COVID-19 trends and mAb referral data from CDPHE helped better understand population-level trends in referral and uptake and where to focus efforts geographically but did not allow for a real-time stratified analysis of vulnerable groups and populations. The data were supplemented with insights from the advisory panel, which included community advocates and regional health connectors with knowledge of community needs, who helped identify underserved groups and populations who may benefit most. The referral data were used to robustly evaluate dissemination impact, beyond simply reporting website views or social media shares.

Another novel contribution of the F2C Framework is an emphasis on the concept of assessing “fit to context” within a given phase to determine readiness to move to the next phase (e.g., readiness to move from conceptualization to design) or need to move back to a prior phase (e.g., emerging evidence from dissemination and impact phases suggests the need for additional design phase activities). While the concept of “fit” or “alignment” is acknowledged as important in D&I (39) (for instance, in the Consolidated Framework for Implementation Research as “compatibility”) (40), what constitutes “fit” between an intervention and its context of expected use has not been comprehensively defined. Operationalizing fit to context at each phase of this project required an iterative understanding of (1) the context in which mAbs were being disseminated; and (2) the extent to which both mAbs themselves and the dissemination strategy aligned with that context. In the Design Phase, this meant identifying mAbs messaging, packaging, and distribution channels aligned with the needs of target audiences. It also meant understanding what mAb referral steps were feasible, acceptable, and appropriate in the common clinical contexts in which people eligible for mAb treatment may be identified.

For both community and provider audiences, the dissemination strategies needed to include education about evidence of mAb effectiveness *and* real-world, practical guidance about how to access and provide mAb treatment. This scientific and policy information changed often, requiring frequent updates. While adaptation to dynamic context is often integral to dissemination and implementation research (41), especially during public health crises (42), it was particularly necessary in this project. In fact, there may be no better descriptor of the COVID-19 pandemic than “dynamic context.” Context changes included rapid-but-rigorous development of COVID-19 treatments and vaccinations, evolution in dominant variants, and often-times weekly changes in public health policy and FDA approvals.

Leveraging well-established partnerships, public health and health system infrastructure, and long-term relationships with community organizations was critical to mAb Colorado’s success. This project would not have been possible without the willingness of multi-sector partners to use and build upon existing system capacity to enhance awareness and access to mAb treatment. For example, both UCHealth’s virtual health center and the CTSA’s clinical and translational research center redirected and adapted resources to increase capacity for mAb referrals, scheduling, and infusion. The ECHO Colorado team served as a major pathway for provider-targeted education and implementation guidance. Project ECHO was a trusted messenger for providers (determined through conceptualization phase provider interviews) with a finely tuned system for marketing, hosting, and evaluating webinars and providing continuing education credit. Our experience was consistent with others nationally, such that Project ECHO proved a useful partner for COVID-19 provider education (43). Responsiveness to dynamic context and the need for rapid dissemination of evidence in a pandemic benefit from the ability to call upon such communication channels and care delivery systems. This project also demonstrated the value that the United States’ national CTSA program offers in terms of adaptive capacity for clinical research, informatics, community engagement, and D&I science that can be called upon in response to public health emergencies (44). Resources should be committed on an ongoing basis to enable rapid responses to future dynamic public health emergencies – that is, we must maintain a “warm base” of infrastructure that can be repurposed for future public health needs.

This paper adds to the growing literature on strategies for making the process of research and implementation of evidence into practice more rapid – including that which emerged in response to COVID-19 (42, 45, 46). In our case, rapid research and implementation meant to undertake the usual research steps in a more efficient manner in order to draw conclusions, implement interventions, and achieve impact more quickly. There are several ways of conceptualizing what makes D&I research more rapid, ranging from the pace of an implementation effort— such as in this project—to the speed with which health care leads to improved clinical outcomes (8). The “rapid” strategies used in this project included (1) bypassing the need for some primary data collection by using “real-world data” gathered in the course of routine care and public health surveillance activities for monitoring geographic need and impact, (2) bypassing the need for creating new distribution channels by leveraging existing relationships between research, clinical and public health practitioners, and health education and communication entities, such as through practice-based research networks, RHCs, and project ECHO, (3) conducting pragmatic and hybrid implementation-effectiveness research, such that by studying effectiveness and implementation in parallel, we were able to quickly understand what works in usual care settings from both perspectives and make adjustments in real time, and (4) efficiently integrating evidence-based practices from many disciplines through use of team science. The mAb Colorado project also included a major emphasis on studying real-world effectiveness of mAbs (47–50) and innovative approaches to assessing fair and equitable treatment allocation (51) leading to significant national policy impact. Other methods for making research more “rapid” used in this project include rapid qualitative analysis (52) used to assess contextual factors important to dissemination strategy design and enactment, as well as real-time evaluation (53) to assess needed adaptations dissemination strategies.

Recently, Proctor and colleagues introduced the FAST Framework to aid in the assessment of the speed of translation in policy and practice (8). Our project reflected several aspects of the FAST Framework—demonstrating a rapid pace of implementation of mAb referral and treatment processes (parameters of speed), faster progression through the F2C framework process phases than might otherwise be done in a non-public health emergency context (effects of speed), having meaningful partnerships and receptive audiences (rate of flow factors), and orientation to urgent need and implementation capacity (accelerators). Proctor and colleagues call for more research on testing speed of adoption, implementation, and impact of rapid methods, using hybrid implementation-effectiveness studies and natural experiments.

There were several limitations to our rapid D4DS approach and our application of the F2C framework. As noted by others (53), a tradeoff in use of rapid evaluation methods in the context of humanitarian emergencies is balancing the need for speed with trustworthiness of the data. While it was helpful to use the real-time COVID-19 epidemiological data to inform decisions related to priority geographic regions and communities for dissemination, the precision of these data is unclear. These data allowed assessment of real-time effectiveness of selected channels, allowing for appropriate pivots. Tracking impact of our dissemination activities was challenging. Although most materials were electronic (enabling tracking reach), we provided print copies of the materials for distribution – which were harder to track. Given the observational nature of this project as a natural experiment, we cannot draw strong conclusions about the causal effect (or proportional effect) of our dissemination activities relative to other historical factors. While we used rigorous causal inference methods in the impact phase, we cannot rule out the effects of factors such as well-known public figures receiving mAbs for COVID-19.

## 5 Conclusion

Rapid methods for designing for dissemination are feasible to accomplish while still using rigorous application of D&I frameworks and methods, including the novel Fit to Context Framework for Designing for Dissemination and Sustainability. The pace at which this project progressed was likely achieved because of four factors: (1) A robust existing CTSA infrastructure including a mature dissemination and implementation research core; (2) Strong, long-term relationships with multi-sector community, health system, and public health partners; (3) A substantial budget for D&I activities made available through supplemental federal funding to our CTSA; and (4) A team with deep clinical and policy knowledge about relevant science and policy decisions—sometimes before they were even public—which allowed us to prepare and be in the position to advise health departments, policy makers, health systems, and community partners.

## Data Availability

The raw data supporting the conclusions of this article will be made available by the authors, with appropriate regulatory approvals.

## Footnotes

^1^https://youtu.be/sRloUq5H-UQ

## Licenses and permissions

Figure 1 was reproduced with permission from Oxford University Press, publishers of the text Dissemination and Implementation Research in Health, 3rd edition. This content is excluded from all forms of open access license, including Creative Commons, and the content may not be reused without the permission of Oxford University Press - details of how to obtain permission can be found at https://global.oup.com/academic/rights/permissions/?cc=gb&lang=en&.

## References

[1] Proctor EK Geng E. A new lane for science. Science. (2021) 374:659. doi: 10.1126/science.abn0184 34735221

[2] Mahase E. Covid-19: FDA authorises neutralising antibody bamlanivimab for non-admitted patients. Br Med J. (2020) 371:m4362. doi: 10.1136/bmj.m4362 33177042

[3] Gottlieb RL Nirula A Chen P Boscia J Heller B Morris J. Effect of bamlanivimab as monotherapy or in combination with etesevimab on viral load in patients with mild to moderate COVID-19: a randomized clinical trial. JAMA. (2021) 325:632–44. doi: 10.1001/jama.2021.0202, PMID: 33475701 PMC7821080

[4] Behr CL Maddox KEJ Meara E Epstein AM Orav EJ Barnett ML. Anti–SARS-CoV-2 monoclonal antibody distribution to high-risk Medicare beneficiaries, 2020-2021. JAMA. (2022) 327:980–3. doi: 10.1001/jama.2022.1243, PMID: 35119452 PMC8904305

[5] Wiltz JL Feehan AK Molinari NM Ladva CN Truman BI Hall J. Racial and ethnic disparities in receipt of medications for treatment of COVID-19—United States, march 2020–august 2021. Morb Mortal Wkly Rep. (2022) 71:96–102. doi: 10.15585/mmwr.mm7103e1, PMID: 35051133 PMC8774154

[6] Baumann AA Shelton RC Kumanyika S Haire-Joshu D. Advancing healthcare equity through dissemination and implementation science. Health Serv Res. (2023) 58:327–44. doi: 10.1111/1475-6773.14175, PMID: 37219339 PMC10684051

[7] Kwan BM Brownson RC Glasgow RE Morrato EH Luke DA. Designing for dissemination and sustainability to promote equitable impacts on health. Annu Rev Public Health. (2022) 43:331–53. doi: 10.1146/annurev-publhealth-052220-112457, PMID: 34982585 PMC9260852

[8] Proctor E Ramsey AT Saldana L Maddox TM Chambers DA Brownson RC. FAST: a framework to assess speed of translation of health innovations to practice and policy. Glob Implement Res Appl. (2022) 2:107–19. doi: 10.1007/s43477-022-00045-4 35669171 PMC9161655

[9] Adsul P Chambers D Brandt HM Fernandez ME Ramanadhan S Torres E. Grounding implementation science in health equity for cancer prevention and control. Implement Sci Commun. (2022) 3:56. doi: 10.1186/s43058-022-00311-4, PMID: 35659151 PMC9164317

[10] Brownson RC Kumanyika SK Kreuter MW Haire-Joshu D. Implementation science should give higher priority to health equity. Implement Sci. (2021) 16:1–16. doi: 10.1186/s13012-021-01097-0 33740999 PMC7977499

[11] Yousefi Nooraie R Shelton RC Fiscella K Kwan BM McMahon JM. The pragmatic, rapid, and iterative dissemination and implementation (PRIDI) cycle: adapting to the dynamic nature of public health emergencies (and beyond). Health Research Policy Syst. (2021) 19:1–10. doi: 10.1186/s12961-021-00764-4 PMC8335455 34348732

[12] Chambers DA Glasgow RE Stange KC. The dynamic sustainability framework: addressing the paradox of sustainment amid ongoing change. Implement Sci. (2013) 8:1–11. doi: 10.1186/1748-5908-8-117 24088228 PMC3852739

[13] Yousefi Rabin BA Brownson RC. Terminology for dissemination and implementation research In: Brownson RC Colditz GA Proctor EK, editors. Dissemination and implementation research in health: translating science to practice. New York, New York: Oxford University Press (2018). 19–45.

[14] Dearing JW Kreuter MW. Designing for diffusion: how can we increase uptake of cancer communication innovations? Patient Educ Couns. (2010) 81:S100–10. doi: 10.1016/j.pec.2010.10.013, PMID: 21067884 PMC3000559

[15] Nooraie RY Kwan BM Cohn E AuYoung M Roberts MC Adsul P. Advancing health equity through CTSA programs: opportunities for interaction between health equity, dissemination and implementation, and translational science. J Clin Transl Sci. (2020) 4:168–75. doi: 10.1017/cts.2020.10 32695484 PMC7348010

[16] Kerkhoff AD Farrand E Marquez C Cattamanchi A Handley MA. Addressing health disparities through implementation science—a need to integrate an equity lens from the outset. Implement Sci. (2022) 17:1–4. doi: 10.1186/s13012-022-01189-5 35101088 PMC8802460

[17] Kwan BM Luke DA Adsul P Koorts H Morrato EH Glasgow RE. Designing for dissemination and sustainability: principles, methods, and frameworks for ensuring fit to context In: Brownson RC Colditz GA Proctor EK, editors. Dissemination and implementation research in health: Translating science to practice. 3rd Edition ed. New York: Oxford University Press (2023)

[18] Dearing JW. Improving the state of health programming by using diffusion theory. J Health Commun. (2004) 9:21–36. doi: 10.1080/10810730490271502 14960402

[19] Bauman AE Nelson DE Pratt M Matsudo V Schoeppe S. Dissemination of physical activity evidence, programs, policies, and surveillance in the international public health arena. Am J Prev Med. (2006) 31:57–65. doi: 10.1016/j.amepre.2006.06.026, PMID: 16979470

[20] Greenhalgh T Robert G Macfarlane F Bate P Kyriakidou O. Diffusion of innovations in service organizations: systematic review and recommendations. Milbank Q. (2004) 82:581–629. doi: 10.1111/j.0887-378X.2004.00325.x, PMID: 15595944 PMC2690184

[21] Hamer MK Alasmar A Kwan BM Wynia MK Ginde AA DeCamp MW. Referrals, access, and equity of monoclonal antibodies for outpatient COVID-19: a qualitative study of clinician perspectives. Medicine. (2022) 101:e32191. doi: 10.1097/MD.0000000000032191, PMID: 36550877 PMC9771255

[22] Kwan BM Sobczak C Beaty L Wynia MK DeCamp M Owen V. Clinician perspectives on monoclonal antibody treatment for high-risk outpatients with COVID-19: implications for implementation and equitable access. J Gen Intern Med. (2022) 37:3426–34. doi: 10.1007/s11606-022-07702-2, PMID: 35790666 PMC9255528

[23] Kwan BM Sobczak C Gorman C Roberts S Owen V Wynia MK. “All of the things to everyone everywhere”: a mixed methods analysis of community perspectives on equitable access to monoclonal antibody treatment for COVID-19. PLoS One. (2022) 17:e0274043. doi: 10.1371/journal.pone.0274043, PMID: 36417457 PMC9683597

[24] Hamer MK Sobczak C Whittington L Bowyer RL Koren R Begay JA. Real-world data to evaluate effects of a multi-level dissemination strategy on access, outcomes, and equity of monoclonal antibodies for COVID-19. J Clin Transl Sci. (2023) 7:e258. doi: 10.1017/cts.2023.679, PMID: 38229899 PMC10789982

[25] Joosten YA Israel TL Williams NA Boone LR Schlundt DG Mouton CP. Community engagement studios: a structured approach to obtaining meaningful input from stakeholders to inform research. Acad Med. (2015) 90:1646–50. doi: 10.1097/ACM.0000000000000794, PMID: 26107879 PMC4654264

[26] Zhou C Crawford A Serhal E Kurdyak P Sockalingam S. The impact of project ECHO on participant and patient outcomes: a systematic review. Acad Med. (2016) 91:1439–61. doi: 10.1097/ACM.0000000000001328, PMID: 27489018

[27] Arora S Kalishman S Thornton K Dion D Murata G Deming P. Expanding access to hepatitis C virus treatment-extension for community healthcare outcomes (ECHO) project: disruptive innovation in specialty care. Hepatology. (2010) 52:1124–33. doi: 10.1002/hep.23802, PMID: 20607688 PMC3795614

[28] Colorado Health Institute. Colorado COVID-19 social distancing index. (2020). Available from: https://www.coloradohealthinstitute.org/research/colorado-covid-19-social-distancing-index (Accessed May 8, 2023)

[29] Aggarwal NR Beaty LE Bennett TD Carlson NE Mayer DA Molina KC. Change in effectiveness of sotrovimab for preventing hospitalization and mortality for at-risk COVID-19 outpatients during an omicron BA. 1 and BA. 1.1-predominant phase. Int J Infect Dis. (2023) 128:310–7. doi: 10.1016/j.ijid.2022.10.002 36229005 PMC9549713

[30] Lum H Kwan B Sobczak C Fish L Keller R Gottsman A. COVID-19 monoclonal antibody (mAb) implementation blueprint. COVID-19 mAb implementation blueprint. (2020). Available at: https://digitalcollections.cuanschutz.edu/work/ns/7faf7c43-1db7-4d5e-a392-204a33760a1f (Accessed May 8, 2023)

[31] Reno J. mAb Colorado communication kit web page. mAb Colorado community messaging materials. Available at: https://digitalcollections.cuanschutz.edu/work/ns/52273633-1c9f-4cb4-b1dc-d63edaa3c668 (Accessed May 8, 2023)

[32] Reno J. mAb Colorado provider kit web page. mAb Colorado provider messaging materials: Available at: https://digitalcollections.cuanschutz.edu/work/ns/645c0544-6b19-42a6-9462-2e055abfe248 (Accessed May 8, 2023)

[33] Goldberg E Kao D Kwan B Patel H Hassell A Zane R. UCHealth’s virtual health center: how Colorado’s largest health system creates and integrates technology into patient care. NPJ Digital Med. (2024) 7:187. doi: 10.1038/s41746-024-01184-8, PMID: 38992097 PMC11239912

[34] Fish LE Bendelow T Gardiner S Wynia MK Kwan BM Hamer MK. A multimodal strategy to improve race/ethnic group equity in administration of neutralizing monoclonal antibody treatment for COVID-19 outpatients. J Clin Transl Sci. (2023) 7:e37. doi: 10.1017/cts.2022.526, PMID: 36845303 PMC9947608

[35] Colorado Department of Public Health and Environment. Amended public health order 21-02 Concerning access to care November 19, 2021 (2021)

[36] Peek CJ Glasgow RE Stange KC Klesges LM Purcell EP Kessler RS. The 5 R's: an emerging bold standard for conducting relevant research in a changing world. Ann Fam Med. (2014) 12:447–55. doi: 10.1370/afm.1688, PMID: 25354409 PMC4157982

[37] Baumann AA Hooley C Kryzer E Morshed AB Gutner CA Malone S. A scoping review of frameworks in empirical studies and a review of dissemination frameworks. Implement Sci. (2022) 17:53. doi: 10.1186/s13012-022-01225-4, PMID: 35945548 PMC9361268

[38] Schlechter CR Reese TJ Wirth J Gibson B Kawamoto K Siaperas T. Rapid-cycle designs to adapt interventions for COVID-19 in safety-net healthcare systems. Transl Behav Med. (2023) 13:389–99. doi: 10.1093/tbm/ibac101, PMID: 36999823 PMC10255772

[39] Lundmark R Hasson H Richter A Khachatryan E Åkesson A Eriksson L. Alignment in implementation of evidence-based interventions: a scoping review. Implement Sci. (2021) 16:1–14. doi: 10.1186/s13012-021-01160-w 34711259 PMC8554825

[40] Damschroder L. The consolidated framework for implementation research (CFIR). Implement Sci. (2022):38–41. doi: 10.4324/9781003109945-11 23663819 PMC3656778

[41] Shelton RC Chambers DA Glasgow RE. An extension of RE-AIM to enhance sustainability: addressing dynamic context and promoting health equity over time. Front Public Health. (2020) 8:134. doi: 10.3389/fpubh.2020.00134, PMID: 32478025 PMC7235159

[42] Eisman AB Kim B Salloum RG Shuman CJ Glasgow RE. Advancing rapid adaptation for urgent public health crises: using implementation science to facilitate effective and efficient responses. Front Public Health. (2022) 10:959567. doi: 10.3389/fpubh.2022.959567, PMID: 36091566 PMC9448975

[43] Moore JD de Leon Gonzalez J Casanova MP Rodgers K Baker RT. An examination of an adapted Project ECHO model series during the COVID‐19 pandemic in Idaho. Pub Health Challenges (2023) 2:e128. 10.1002/puh2.128 PMC12039751 40496783

[44] Volkov BB Ragon B Doyle JM Bredella MA. Adaptive capacity and preparedness of clinical and translational science award program hubs: overview of an environmental scan. J Clin Transl Sci. (2023) 7:e31. doi: 10.1017/cts.2022.400, PMID: 36845304 PMC9947610

[45] Smith J Rapport F O’Brien TA Smith S Tyrrell VJ Mould EVA. The rise of rapid implementation: a worked example of solving an existing problem with a new method by combining concept analysis with a systematic integrative review. BMC Health Serv Res. (2020) 20:449. doi: 10.1186/s12913-020-05289-0, PMID: 32438909 PMC7240003

[46] Quanbeck A Hennessy RG Park L. Applying concepts from "rapid" and "agile" implementation to advance implementation research. Implement Sci Commun. (2022) 3:118. doi: 10.1186/s43058-022-00366-3, PMID: 36335373 PMC9636827

[47] Wendel SK Wogu AF Carlson NE Beaty L Bennett TD Bookman K. Effectiveness of subcutaneous monoclonal antibody treatment in emergency department outpatients with COVID-19. J Am College Emerg Physicians Open. (2024) 5:e13116. doi: 10.1002/emp2.13116, PMID: 38384380 PMC10879902

[48] Roberts SC Jolley SE Beaty LE Aggarwal NR Bennett TD Carlson NE. Association between monoclonal antibody therapy, vaccination, and longer-term symptom resolution after acute COVID-19. J Med Virol. (2024) 96:e29541. doi: 10.1002/jmv.29541, PMID: 38516779 PMC10963040

[49] Aggarwal NR Beaty LE Bennett TD Carlson NE Davis CB Kwan BM. Real-world evidence of the neutralizing monoclonal antibody sotrovimab for preventing hospitalization and mortality in COVID-19 outpatients. J Infect Dis. (2022) 226:2129–36. doi: 10.1093/infdis/jiac206, PMID: 35576581 PMC10205600

[50] Wynia MK Beaty LE Bennett TD Carlson NE Davis CB Kwan BM. Real-world evidence of neutralizing monoclonal antibodies for preventing hospitalization and mortality in COVID-19 outpatients. Chest. (2023) 163:1061–70. doi: 10.1016/j.chest.2022.10.020, PMID: 36441040 PMC9613796

[51] Roberts SC Jolley SE Beaty LE Aggarwal NR Bennett TD Carlson NE. Association between monoclonal antibody therapy, vaccination, and longer‐term symptom resolution after acute COVID‐19. J Med Virol. (2024) 96:e29541. 10.1002/jmv.29541 PMC10963040 38516779

[52] Lewinski AA Crowley MJ Miller C Bosworth HB Jackson GL Steinhauser K. Applied rapid qualitative analysis to develop a contextually appropriate intervention and increase the likelihood of uptake. Med Care. (2021) 59:S242–51. doi: 10.1097/MLR.0000000000001553, PMID: 33976073 PMC8132894

[53] McNall M Foster-Fishman PG. Methods of rapid evaluation, assessment, and appraisal. Am J Eval. (2007) 28:151–68. doi: 10.1177/1098214007300895

